# Transcranial Magnetic Stimulation Applications in the Study of Executive Functions: A Review

**DOI:** 10.1002/brb3.70099

**Published:** 2024-11-25

**Authors:** Muyu Chen, Guang Zhao, Li Peng

**Affiliations:** ^1^ Department of Military Psychology, School of Psychology Army Medical University Chong‐Qing China; ^2^ Department of Medical Services Xingcheng Sanatorium of PLA Joint Logistics Support Force Huludao Liaoning China

**Keywords:** cognition enhancement, cognitive flexibility, executive functions, transcranial magnetic stimulation

## Abstract

**Purpose:**

Executive functions (EFs) are a set of advanced cognitive functions essential for human survival and behavioral regulation. Understanding neurophysiological mechanisms of EFs as well as exploring methods to enhance them are still challenging problems in cognitive neuroscience. In recent years, transcranial magnetic stimulation (TMS) has been widely used in the field of EF research and has made notable progress. This article aimed to discuss the impact of TMS technology on EF research from both basic and applied research perspectives.

**Methods:**

We searched for literature on TMS and EFs published in the last decade (2013–2023) and reviewed how TMS has been applied in the field of EF.

**Findings:**

We found that the combination of TMS with neuroimaging techniques was helpful in exploring the brain mechanisms of EFs and investigating the executive dysfunctions caused by other neuropsychiatric disorders. Moreover, TMS could be considered as one of the most important techniques to enhance EFs among patient populations, even healthy people, with high safety and credibility. Meanwhile, we discussed the application of TMS in the research of EFs and made suggestions for future research directions. We suggested that a multidisciplinary structure of methods such as epigenetics and endocrinology could be integrated with TMS for investigating deeper in EF domains, and a substantial number of high‐quality clinical studies are required to further elucidate the effects of TMS on EFs.

**Conclusions:**

TMS holds great promise for gaining insight into investigating the neural mechanisms of EFs and improving executive functions among different populations.

## Graphical abstract

The structure of this review is as follows. First, we introduced what is the executive functions (EFs). Then, we reviewed the progress of transcranial magnetic stimulation (TMS) in EFs basic and applied research in the last decade. And we prospected the future of TMS in EFs research.

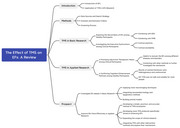

## 1 Introduction

### 1.1 Introduction of Executive Functions

Executive functions (EFs), also known as cognitive control (CC), encompass a set of advanced cognitive abilities that enable humans to set goals, adapt to new environments, manage social interactions, and regulate their behaviors according to internal goals and constraints (Diamond 2013). Generally, the core components of EFs include working memory, inhibitory control, and cognitive flexibility, along with reasoning, problem‐solving, and planning (Cristofori, Cohen‐Zimerman, and Grafman 2019). EFs can be further categorized into “hot” EFs, which involve processing information related to rewards, emotions, and motivation, including components such as emotional regulation and reward processing, and “cold” EFs, which are limited to the processing of cognitive information, corresponding to the narrow definition of EFs (Salehinejad et al. 2021). EFs engage multiple parts of the brain, with the executive control network (ECN, including dorsolateral prefrontal cortex, posterior parietal cortex, anterior cingulate cortex, etc.) (Friedman and Robbins 2022) and default mode network (DMN, including medial prefrontal cortex, posterior cingulate cortex, angular gyrus, etc.) (Zhang and Volkow 2019) playing particularly prominent roles. And as the primary node of the ECN and DMN, the prefrontal cortex (PFC) adjusts cognitive abilities to meet various demands by regulating cortico‐cortical, cortico‐striatal, and cortico‐limbic connections (Luciana and Collins 2022). The factors affecting EFs include age, gender, emotion, stress, sleep, etc. (Ganesan and Steinbeis 2022). High levels of EFs contribute to good emotional regulation and cognitive abilities, which are crucial for individual growth and development, interpersonal communication, and academic research (Horowitz‐Kraus, Randell, and Morag 2023). Conversely, many neuropsychiatric disorders are characterized by deficits in EFs (Jones and Graff‐Radford 2021). Therefore, studying the neuropsychological foundations of EFs and seeking reasonable means to enhance them is one of the pivotal issues in cognitive neuroscience research.

### 1.2 Application of Transcranial Magnetic Stimulation in Executive Functions Research

As one of the noninvasive brain stimulation (NIBS) techniques that uses electromagnetic induction to generate currents and transiently alter the activity of the cortex, transcranial magnetic stimulation (TMS) has increasingly garnered attention from researchers for its therapeutic and other utilizations on EFs (Lefaucheur 2019). TMS encompasses various modalities such as single‐pulse TMS (spTMS), paired‐pulse TMS (ppTMS), and repetitive TMS (rTMS) (for more details on these modalities, see Table 1) (Suppa, Asci, and Guerra 2022). TMS paradigms with a stimulation frequency of less than 5 Hz are referred to as low‐frequency TMS (LF‐TMS), which has an inhibitory effect (Cox et al. 2023), while those with a frequency of more than 5 Hz are called high‐frequency TMS (HF‐TMS), which has an excitatory effect (Eysel and Jancke 2024). Since its first clinical application in 1985 (Barker, Jalinous, and Freeston 1985), TMS technology has been widely used in the diagnosis and treatment of various neurological diseases [such as Parkinson's disease (PD) (He et al. 2022), mild traumatic brain injury (mTBI) (Buhagiar et al. 2020), and stroke (Gao et al. 2023)] and mental disorders [such as depression (Cappon et al. 2022), obsessive‐compulsive disorder (Fitzsimmons et al. 2022), bipolar disorder (Hsu et al. 2024), and primary sleep disorder (Nardone et al. 2020)], yielding favorable therapeutic outcomes. Additionally, TMS plays a significant role in understanding the activation/inhibition and plasticity, connectivity, and excitability of the cerebral cortex, as well as in preoperative functional localization (Siebner et al. 2022), and is considered a promising auxiliary diagnostic tool for various neuropsychiatric disorders (Chou et al. 2022, Cao et al. 2021). The application of TMS in EF research primarily includes: (1) Exploring the neural basis of EFs, such as the specific role of a particular brain structure in the EF process; (2) Combining with EF‐related neuropsychological tests as a tool to assess cortical changes under different conditions (e.g., patients vs. healthy controls; preintervention vs. postintervention), such as cortical plasticity and excitability; (3) Serving as a tool to improve participants’ EFs (e.g. enhancing EFs of individuals with insomnia by regulating their sleep homeostasis and plasticity). However, despite the abundance of related research, there are few reviews that systematically analyze and synthesize the work of previous studies during the last decade.

**TABLE 1:** Comparison of common TMS modalities.

Modalities	Variants	Definition	Commonly usage
spTMS	—	spTMS means that using TMS equipment to generate a short‐lived impulse in the cortex to trigger rapid depolarization of neurons.	Diagnostic biomarkers and neuroscience research probes (RMT, MEP, CSP, etc.)
ppTMS	PAS	ppTMS refers to that two TMS pulses, conditioning stimulus and test stimulus, are delivered with different interstimulus intervals and intensities in succession. The modulation of the test stimulus by the conditioning stimulus usually serves as the primary measure of interest.	Diagnostic biomarkers and neuroscience research probes (LICI/LICF, SICI/SICF, SAI/LAI, LTP/LTD, etc.)
rTMS	iTBS, cTBS, QPS, etc.	rTMS is a TMS modality that uses repetitive magnetic pulses (a combination of ≥ 3 pulses of a specific intensity, with a frequency of at least 0.5 pulses/s) applied to the scalp to modulate the excitability of cortical neurons.	Clinical and therapeutic practices (adjunctive therapies, neuromodulatory treatment, cognition enhancement for patient and healthy populations)

*Note*: Summarized from reviews by Burke, Fried, and Pascual‐Leone (2019) and Sun et al. (2023).

Abbreviations: CSP: cortical silent potential; cTBS: continuous theta burst stimulations; iTBS: intermittent theta burst stimulations; LICI/LICF: long‐interval intracortical inhibition/facilitation; LTP/LTD: long‐term potentiation/depression; MEP: motor‐evoked potential; PAS: paired associative stimulations; ppTMS: paired‐pulse transcranial magnetic stimulations; QPS: Quadri‐pulse stimulations; RMT: rest motor threshold; rTMS: repetitive transcranial magnetic stimulations; SAI/LAI: short/long‐latency afferent inhibition; SICI/SICF: short‐interval intracortical inhibition/facilitation; spTMS: single‐pulsed transcranial magnetic stimulations.

### 1.3 Structure of the Article

Thus, this article aimed to explore the role of TMS in EF research by examining the research outcomes from the last decade (2013–2023). We discussed the application of TMS in basic EF research, the impact of TMS as an intervention on human EFs, the future of TMS application in the field of EFs, and eventually provided some recommendations for following research directions.

## 2 Methods

### 2.1 Data Sources and Search Strategy

M.C. and L.P. conducted online search on Web of Science, Pubmed, Embase, Cochrane, CNKI, Wanfang, and other online databases independently in preparing this review. Using search on Pubmed as an example, we developed the following search formula:

1. TS = (“executive function” OR “working memory” OR “cognitive flexibility” OR “inhibitory control” OR “planning” OR “problem solving” OR “reasoning” OR “cognitive control” OR “Executive Functions” OR “Function, Executive” OR “Functions, Executive” OR “Executive Control” OR “Executive Controls”)
2. TS = (“Transcranial Magnetic Stimulation” OR “Magnetic Stimulation, Transcranial” OR “Magnetic Stimulations, Transcranial” OR “Stimulation, Transcranial Magnetic” OR “Stimulations, Transcranial Magnetic” OR “Transcranial Magnetic Stimulations” OR “TMS” OR “Transcranial Magnetic Stimulation, Single Pulse” OR “Transcranial Magnetic Stimulation, Paired Pulse” OR “Transcranial Magnetic Stimulation, Repetitive”)
3. #1 AND #2

### 2.2 Inclusion and Exclusion Criteria

#### 2.2.1 Inclusion Criteria

(1) Articles published between January 2013 and December 2023 and (2) articles concerning the relationship between TMS and EFs.

#### 2.2.2 Exclusion Criteria

(1) Articles not published in English or Chinese, (2) TMS was not the primary intervention method, and (3) EFs were not the primary or secondary outcomes.

### 2.3 Screening Process

First, each reviewer independently assessed the titles and abstracts of the available records to exclude those papers that met the exclusion criteria. Then, the selected papers underwent a detailed full‐text review for ensuring that they met the inclusion criteria.

## 3 The Role of TMS in Basic Research: A Significant Probing Tool

### 3.1 Healthy Participants: Exploring the Neural Basis of Executive Functions

In research involving EFs among healthy participants, TMS is commonly employed to inhibit and/or activate specific brain regions, primarily for investigating the roles of different brain areas. It is frequently combined with functional magnetic resonance imaging (fMRI) for this purpose. It is worth noting that, to ensure precision, the localization of TMS in most basic research studies is often achieved through fMRI‐guided neuronavigation systems; additionally, before and after stimulation, fMRI can monitor changes in blood oxygen level–dependent (BOLD) signals to reflect the level of activation or inhibition of specific brain regions, thereby investigating their roles in specific cognitive processes (Mizutani‐Tiebel et al. 2022). Therefore, TMS combined with fMRI is quite widely used for the study of neural mechanisms involved in EFs. In studies of working memory, Morgan et al. (2013) hypothesized that the right parietal cortex (r‐PC) and the left inferior frontal gyrus (l‐IFG) played a collaborative role in processing spatial and visual working memory. The results of experiments using continuous theta burst stimulation (cTBS) on these areas validated this hypothesis. Yue and Martin (2022), targeting the two theoretical models of phonological working memory (the embedded‐process model vs the buffer model), used spTMS to stimulate the superior temporal gyrus (STG, assumed to be the speech perception area according to the embedded‐process model) and the supramarginal gyrus (SMG, assumed to be the buffer area according to the buffer model), respectively. The results showed that online TMS targeted at the SMG reduced the performance of working memory in the recognition of nonwords, while stimulation of the STG did not produce a significant effect. This outcome supported the buffer model hypothesis and the role of the SMG in maintaining phonological representations. Kiyonaga et al. (2021) investigated the impact of parietal lateralization on working memory and attention using fMRI‐guided TMS. Their research revealed that although the r‐PC had a more significant effect on working memory and attention, performance in working memory and attention only declined when the overall activity of the parietal lobe was affected. In studies on inhibitory control, Muhle‐Karbe, Jiang, and Egner (2018) explored the lateral prefrontal cortex (LPFC) and found that TMS eliminated the adaptive fluctuations of attentional focus in participants during the Stroop task, thereby validating the core role of this region in top‐down inhibitory control. Osada et al. (2019) targeted the intraparietal sulcus (IPS) with online TMS and found that stimulation of this area prolonged the response time in the stop‐signal task, suggesting the key role of the IPS in the process of inhibitory control. Parris et al. (2021) questioned the role of the left dorsolateral prefrontal cortex (l‐DLPFC) in inhibiting conflict during inhibitory control, and results of their experiments combining rTMS with the Stroop task suggested that although the task response time was reduced, stimulation of the l‐DLPFC did not affect the control of responses, semantics, or overall conflict. Therefore, it was suggested that the l‐DLPFC might serve as a compensatory function in the Stroop task. In studies targeting other EFs, such as planning, Kaller et al. (2013) applied cTBS over bilateral DLPFCs and discovered that there was indeed a functional dissociation between the two sides during the Tower of London task. Due to its excellent discriminative ability for brain functional networks, fMRI can also be used to analyze the impact of interactions between different brain networks on EFs. For instance, Webler et al. (2022) found, through offline spTMS of the l‐DLPFC during the execution of an N‐back working memory task, that with the increase in cognitive load under the influence of spTMS, the activation nodes of the fronto‐parietal network (FPN) and the deactivation nodes of the DMN also increased. This finding provided evidence for the interaction between FPN and DMN under working memory task conditions.

Furthermore, the combination of TMS with electroencephalography (EEG) often provides novel insights to the study of EFs. EEG can record the electrical signals of neurons that are inhibited or activated by TMS. Although its spatial resolution is relatively lower compared to fMRI, EEG has higher temporal resolution and is typically used to analyze the time‐locked dynamics of event‐related potentials (ERP) and neural oscillations (Hernandez‐Pavon et al. 2023). For instance, Li et al. (2017) used rTMS to stimulate the superior parietal lobule (SPL) in participants during a delayed recognition working memory task, simultaneously recording changes in event‐related spectral perturbations (ERSP) in the *θ* and *α* frequency bands. They found that rTMS enhanced interregional phase synchronization in the *θ* band of EEG between the left superior parietal area and the left frontal area, as well as in the *α* band between the left superior parietal area and both frontal areas. It suggested that rTMS applied over the parietal lobe may indirectly affect the frontal region through the fronto‐parietal pathway, thereby influencing working memory. In addition, TMS can also be used as a tool to measure cortical excitability in conjunction with EEG. As demonstrated by Cespon et al. (2022), after recording the EEG activity of young and elderly participants during a Simon task, they applied spTMS to record global average field power and TMS‐evoked potentials (TEP) in the participants. This was done to analyze the relationship between cortical excitability, inhibitory control, and underlying neural activity. Results of the experiment suggested that age‐related changes in cortical excitability could serve as a representation of functional dysregulation associated with physiological aging.

### 3.2 Clinical Participants: Executive Dysfunctions Caused by Disorders

Compared to healthy populations, TMS studies on EF mechanisms in patients are primarily focused on exploring executive dysfunctions and their neural underpinnings caused by neurological and psychiatric diseases. Therefore, TMS research in this direction is centered around the measurement of cortical excitability and/or cortical plasticity, typically involving the combination of EEG. The primary indicators of cortical excitability mainly include long‐interval intracortical inhibition/facilitation (LICI/LICF), short‐interval intracortical inhibition/facilitation (SICI/SICF), cortical silent period (CSP), short‐latency afferent inhibition (SAI), etc., which are mainly measured through spTMS and ppTMS (Suppa, Asci, and Guerra 2022). In contrast, indicators of cortical plasticity mainly include long‐term potentiation (LTP) and depression (LTD), which are primarily induced through PAS (Kirkovski et al. 2023) (see Table 2). For example, Hayashi et al. (2018) used spTMS and ppTMS to assess cortical excitability in chronic phase patients with diffuse axonal injury (DAI) caused by TBI patients and healthy controls. They also employed a series of neuropsychological tests to evaluate their EFs. The results showed that the SICI values were higher in the DAI group compared to the control group, and the performance in neuropsychological tests was also worse, suggesting that there was a pathological enhancement of inhibitory control abilities in the chronic phase DAI patients. Using a similar experimental paradigm to study Huntington's disease patients, Kamble et al. (2018) found that the SICI values in Huntington's disease patients were significantly lower than those in the control group, indicating that their inhibitory control abilities were impaired. Li et al. (2022) employed TMS to assess the relationship between limbic structures and DLPFC in 40 patients with major depressive disorder (MDD) and found that the glucose metabolism of the participants’ limbic structures was significantly associated with the LICI of the DLPFC. Noda et al. (2018) observed that, compared to healthy controls, the SAI evoked in the DLPFC of patients with schizophrenia was significantly reduced, while there was no significant difference in the SAI of the primary motor cortex (M1). This may reflect a cholinergic deficit in the PFC of schizophrenia patients, which contributes to their executive dysfunctions. A study aimed at investigating the inhibitory control abilities of patients with alcohol use disorder (AUD) and their TEP showed that both the neuropsychological test performance and TEP of AUD patients indicated significant impairment in inhibitory control abilities (Quoilin et al. 2018). These results indicated that the neural basis of changes in inhibitory control abilities in AUD patients might involve certain motor components. Another study on binge‐eating disorder (BED) found that the CSP values in BED patients were significantly longer than those in the control group and were positively correlated with the response time in the GO/NO‐GO task, which suggests that the binge‐eating behavior in BED patients might be due to a higher threshold for inhibition within the cortex (Antunes et al. 2020). These aforementioned studies suggest that TMS can be an efficacious adjunct to conventional neuropsychological tests in elucidating the mechanisms underlying EFs in clinical populations; moreover, TMS might have the potential to offer valuable insights into the neural underpinnings of EFs.

**TABLE 2:** Common measurement index of TMS.

Aim	Index	Measurement methods	Meaning
Basic elements	RMT	Minimal stimulus intensity leading to the appearance of a “minimal motor response” in the resting muscle during the application of a single TMS pulse in the motor cortex	Reflecting cortical excitability
	MEP	Suprathreshold TMS pulse was used upon the motor cortex, thereby resulting in activation of peripheral muscles innervated by the stimulated cortical area.	Reflecting corticospinal excitability
Cortical excitability	LICI	Two consecutive suprathreshold stimuli were delivered at certain time intervals(usually between 50 and 200 ms)	Reflecting GABABR‐mediated neurotransmitter transmission
	SICI	The MEP was suppressed by a subthreshold conditioned stimulus 2–3 ms after the suprathreshold stimulus	Reflecting GABAAR‐mediated neurotransmitter transmission
	CSP	TMS was performed during the tonic contraction phase of the target muscle	Reflecting GABABR‐mediated neurotransmitter transmission
	SAI	After 20–25 ms of combined median nerve electrical stimulation and TMS stimulation over the M1 area, the median nerve electrical stimulation of the M1 area was applied again	Reflecting cholinergic and GABA circuits
Cortical Plasticity	LTP	iTBS (2 s of stimulation per 10 s for approximately 180–200 s) was applied for induction	Reflecting glutamatergic system regulation
	LTD	cTBS (continuous stimulation for 40–60 s) was applied for induction	Reflecting GABAergic system regulation

*Note*: Summarized from reviews by Cao et al. (2021) and Darmani et al. (2022).

Abbreviations: CSP: cortical silent potential; cTBS: continuous theta burst stimulations; GABA: γ‐aminobutyric acid; GABAAR/GABABR: GABA A/B receptor; iTBS: intermittent theta burst stimulations; LICI: long‐interval intracortical inhibition; LTP/LTD: long‐term potentiation/depression; MEP: motor‐evoked potential; RMT: rest motor threshold; SAI: short‐latency afferent inhibition; SICI: short‐interval intracortical inhibition.

## 4 Applied Research: Promoting Cognition Recovery and Enhancement

### 4.1 Clinical Participants: Promising Adjunctive Therapeutic Means

In the clinical practice of TMS for the treatment of neuropsychiatric diseases, researchers have particularly focused on the impact of this technology on patients’ EFs. Early on, as a novel adjunctive treatment, researchers needed to rule out any detrimental effects of TMS on patients’ cognition. For instance, a study conducted by Schulze et al. (2016) suggested that rTMS over the dorsomedial prefrontal cortex (DMPFC) did not have a significant negative impact on the performance of the Stroop task and Trail Making Test (TMT) in patients with treatment‐resistant depression (TRD). As TMS gradually gained traction in clinical practice and its safety was recognized (see Table 3 for commonly used TMS protocols for the treatment of neuropsychiatric disorders), the question of whether it enhanced EFs emerged as a hot topic of clinical research: Multiple meta‐analyses have pointed out that rTMS not only treated symptoms of schizophrenia (Sciortino et al. 2021), attention‐deficit/hyperactivity disorder (Chen et al. 2023), stroke (Gao et al. 2023), Alzheimer's disease (AD), and/or mild cognitive impairment (MCI) (Chou, That, and Sundman 2020), but also promoted the recovery of their EFs. Studies by Cristancho et al. (2020) and Corlier et al. (2020), respectively, using rTMS and intermittent TBS (iTBS) in elderly patients with MDD, both demonstrated significant improvements in patients’ inhibitory control abilities following treatment. Ameis et al. (2020) randomly administered 20 Hz rTMS (or sham stimulation) targeting the DLPFC for 4 weeks to 40 autism patients with executive dysfunction. They found that among patients with poorer baseline EFs, the group receiving active rTMS showed more significant improvements in EFs compared to the sham stimulation group. A pilot study among patients with post‐concussion syndrome following mTBI showed that 1 Hz rTMS applied over the r‐DLPFC could improve patients’ working memory. Han et al. studied patients with obstructive sleep apnea syndrome (OSA) and found that iTBS effectively could improve working memory in OSA patients (Han et al. 2023). Furthermore, there is research evidence supporting the notion that TMS can enhance EFs in psychiatric disorders such as post‐traumatic stress disorder (Nursey et al. 2020), anorexia nervosa (Dalton et al. 2020), bipolar disorder (Rostami et al. 2022), and substance abuse (Harmelech, Hanlon, and Tendler 2023).

**TABLE 3:** Common TMS protocols for the recovery and enhancement of EFs in clinical population.

EFs domains	Disorders	Modalities	Targets	Courses
Working memory	SCZ	HF‐rTMS or iTBS	Bilateral or left DLPFC	2–4 weeks
	mTBI	HF‐rTMS	Left DLPFC	4 weeks
		LF‐rTMS	Right DLPFC	6 weeks
	MDD	HF‐rTMS	Left DLPFC	4 weeks
		iTBS	Bilateral DLPFC	2 weeks
	BD	HF‐rTMS	Left DLPFC	4 weeks
	PTSD	HF‐rTMS	Bilateral DLPFC	4 weeks
	AD	rPAS	Bilateral DLPFC	2 weeks
Inhibitory control	AUD	HF‐rTMS	Left DLPFC	4 weeks
	BD	HF‐rTMS	Left DLPFC	4 weeks
	MDD	iTBS	Bilateral DLPFC	4 weeks
		HF‐rTMS	Left DLPFC	2 weeks
Cognitive flexibility	MDD	HF‐rTMS	Left DLPFC or Bilateral DMPFC	2–4 weeks
	ASD	iTBS	pSTS	1 week

*Note*: Summarized from guidelines by Lefaucheur et al. (2020).

Abbreviations: AD: Alzheimer's disease; ASD: autism spectrum disorder; AUD: alcohol use disorder; BD: bipolar disorder; DLPFC: dorsolateral prefrontal cortex; DMPFC: dorsomedial prefrontal cortex; HF‐rTMS: high frequency repetitive transcranial magnetic stimulation; iTBS: intermittent theta burst stimulation; LF‐rTMS: low frequency repetitive transcranial magnetic stimulation; MDD: major depressive disorder; mTBI: mild traumatic brain injury; pSTS: posterior superior temporal sulcus; PTSD: posttraumatic stress disorder; rPAS: repetitive paired associative stimulation; SCZ: schizophrenia.

However, some studies have yielded different results: A meta‐analysis conducted by Martin et al. (2016) published in 2016 discussed whether rTMS has a cognitive‐enhancing effect on patients with neuropsychiatric diseases. The paper included 30 randomized controlled trials (RCTs), but it only found a moderate level of improvement in working memory in three studies targeting patients with schizophrenia (Hedge's *g* = 0.507, 95% CI: 0.183 to 0.831). Another meta‐analysis published in 2023, which included 174 RCTs and 7905 patients, indicated that compared to the sham stimulation group, rTMS targeted at the l‐DLPFC had a smaller effect size on CC (Hedge's *g* = −0.230, 95% CI: −0.371 to −0.088) and working memory (Hedge's *g* = −0.198, 95% CI: −0.392 to −0.004) in patients with neuropsychiatric diseases (Kan et al. 2023). Additionally, a meta‐analysis including 12 RCTs on the effects of rTMS on improving cognitive function in patients with PD showed that rTMS had no statistically significant short‐term impact on the working memory of patients (SMD = 0.05, 95% CI: −0.25, 0.35) (He et al. 2022). Reasons for these heterogeneous results above may be attributed to several factors: (1) Small numbers of studies and sample sizes may increase the risk of bias in the research outcomes; (2) variations in the TMS stimulation protocols (rTMS vs. TBS), stimulation targets (most commonly the DLPFC, but also other areas), stimulation frequencies (LF‐TMS vs. HF‐TMS, iTBS vs. cTBS), stimulation courses (1 week, 2 weeks, 4 weeks, or even longer), assessment methods (questionnaires, neuropsychological tests, etc.), and follow‐up periods may lead to inconsistent study results; (3) some studies exhibited lower quality, including those that failed to account for confounding variables such as patient demographics, medication status, etc., which may have reduced the reliability of their results.

In recent years, researchers have broadened their methodology to evaluating the improvement in EFs following TMS intervention, moving beyond traditional questionnaires and/or neuropsychological tests, and have also incorporated fMRI or EEG to investigate the potential mechanisms underlying the enhancement of EFs. For instance, Chen et al. (2022) employed fMRI and proton magnetic resonance spectroscopy (MRS) to assess brain functional connectivity and related metabolite levels before and after a 2‐week TMS intervention in patients with depression; Kumar et al. (2023) applied PAS for a 2‐week intervention in 32 AD patients and combined PAS with EEG to assess the plasticity of DLPFC and *θ*–*γ* coupling in the participants during the working memory task. This exploration into the mechanisms has also led to advancements in clinical practice: a study focused on the right IFG in nicotine‐dependent patients alternated between iTBS and cTBS as intervention methods. Their findings suggested that iTBS might be more beneficial for improving patients’ inhibitory control abilities (Newman‐Norlund et al. 2020).

### 4.2 Healthy Participants: Conflicting Cognition Enhancement Effects

Owing to ethical considerations, TMS interventions aimed at enhancing EFs in healthy participants are more prone to raise controversies compared to those in patient populations. Moreover, a limited number of studies have shown considerable heterogeneity in their results: for example, iTBS over the left prefrontal cortex (l‐PC) (600 pulses/session, 5 sessions/week, 1 week/course) may enhance an individual's working memory (Deng et al. 2022), while TMS over the left cerebellum poses a risk of impairing working memory (Viñas‐Guasch et al. 2022). Pulopulos et al. (2022) used HF‐rTMS (20 Hz) to stimulate r‐DLPFC of participants, which was found to be helpful in enhancing reactive control abilities; however, another study by Friehs et al. (2023) did not observe a reduction in reactive control when they used 1 Hz rTMS to stimulate the same area. Meta‐analysis results also differed between de Boer et al. and Xu et al.: the former included 63 studies on the effects of NIBS on the EFs of healthy participants and found that only HF‐TMS could improve the performance on inhibitory control‐related tasks (de Boer et al. 2021), whereas the latter, which analyzed 24 studies on HF‐TMS, pointed out that various EFs, including working memory and cognitive flexibility (not just inhibitory control), were enhanced in participants after the intervention (Xu et al. 2023). In addition to the possible reasons discussed in the previous section, the large heterogeneity in the results of studies on healthy participants may also be attributed to the number of intervention sessions and the timing of assessment after the intervention. Single‐session TMS interventions, whether inhibitory or excitatory, may have limited effects on achieving “cognitive enhancement,” and immediate assessments following the application of TMS (especially inhibitory TMS) could lead to the conclusion that TMS impairs EFs. For instance, Liu et al. (2021) assessed cognitive flexibility immediately after stimulating l‐DLPFC of participants with 1 Hz rTMS once and found that the participants performed worse on the task after the intervention, thereby concluding that “Low‐frequency rTMS of the left DLPFC can cause decline of cognitive flexibility in executive function.” However, as a commonly used intervention paradigm for insomnia (Lanza et al. 2023) and recommended by relevant guidelines (Wang 2024), this protocol might not be simply summarized as “impairing” to an individual's cognitive flexibility; in other words, if the same protocol was used for multiple sessions in individuals with insomnia and their cognitive flexibility was assessed the day after (or even later) the intervention, perhaps the opposite conclusion would be drawn.

It is particularly noteworthy that in recent years, a method called “do‐it‐yourself (DIY) neurostimulation” has gradually emerged (Fisicaro et al. 2020). Individuals usually learn how to conduct TMS therapy by watching related treatment videos online and purchasing the required components to assemble their own TMS device (Wexler 2022). Nevertheless, considering the potential risks of TMS and the complexity of its device structure, individuals cannot guarantee the quality and safety of their DIY TMS devices. They also cannot ensure that these devices produce the correct magnetic field strength, nor can they ensure the standardization of DIY neurostimulation (Zuk et al. 2018). Therefore, the International Federation of Clinical Neurophysiology (IFCN) was against the practice of DIY TMS (Antal et al. 2022).

## 5 Prospect: Future Has Arrived

### 5.1 Basic Research: How to Investigate Executive Functions Deeper?

Currently, research on TMS involving EF mechanisms primarily utilizes the combination of EEG and/or fMRI. Applying other neuroimaging techniques like functional near‐infrared spectroscopy (fNIRS) might be helpful for apprehending the EFs mechanisms more deeply. Despite the fact that the literature is relatively small, there is some evidence showing that combining TMS and fNIRS to measure or enhance participants’ EFs is promising. Whether in screening the executive dysfunction patients (Liu et al. 2023) or evaluating the behavioral outcomes of different intensities of iTBS (Zhang et al. 2022), fNIRS has preliminarily demonstrated its potential in the field of EFs research combined with TMS. Researchers can explore the combination of TMS with more neuroimaging techniques in order to more comprehensively reflect the mechanism of EFs.

Moving forward, researchers can build upon the foundation of TMS combined with neuroimaging to explore a wider range of potential mechanisms influencing individual EFs using additional methodologies. For example, it is generally believed that LTP and LTD depend on the changes in the levels of synaptic glutamate and *γ*‐aminobutyric acid (GABA) (Rozov, Valiullina, and Bolshakov 2017, Wu et al. 2022), respectively. Hence, if it were feasible to directly measure the concentrations of these neurotransmitters simultaneously with the application of TMS using other methods, more compelling results might be yielded.

Furthermore, other hormones may also play a role in affecting TMS outcomes. To study the impact of endogenous estrogen on cortical plasticity, Chung et al. (2019) had female participants undergo assessments during their menstrual cycle (high‐ and low‐estrogen phases), with male participants serving as controls, and their results showed that women in the high‐estrogen phase showed greater plasticity in the DLPFC. This not only indicated that individual cortical plasticity might undergo periodic changes but also suggested that when using TMS for related research, it might be necessary to consider the timing to achieve more favorable outcomes.

Genetic studies may also provide additional insights into the mechanisms by which TMS affects individual EFs: individuals carrying a certain allele (BDNF Val66Met) associated with different brain‐derived neurotrophic factors may exhibit enhanced selection bias under the influence of r‐DLPFC (Tulviste et al. 2019); moreover, participants with high dopamine signaling transmission are more likely to benefit from rTMS in terms of improving working memory compared to those with low dopamine signaling transmission (Hong et al. 2022).

In addition, animal experiments may also contribute to understanding the anatomical and functional changes in the brain after TMS. For example, results of Zhang et al. (2022) suggested that iTBS can enhance working memory in rats and increase cross‐brain network connectivity between the ventral hippocampus and the medial prefrontal cortex.

In summary, integrating neuroendocrinology, epigenetics, and other multidisciplinary methods with TMS, while also establishing more valuable animal models, represents a potential avenue for future in‐depth research on EFs.

### 5.2 Applied Research: How to Improve Executive Functions More Effectively?

In the clinical practice of TMS, one of the most critical challenges to overcome might be how to enhance the precision of localization, as accurate targeting is the essential foundation for ensuring the effectiveness of the intervention (Polania, Nítsche, and Ruff 2018). Although the use of neuronavigation and computational modeling can individually establish brain structure data for each participant, in clinical conditions, due to considerations of cost and feasibility, therapists often prefer to use the international 10–20 system or locate based on anatomical landmarks. Therefore, developing a simple, practical, and yet accurate method of TMS localization is important for enhancing individual EFs and cannot be overlooked.

Developing more TMS protocols specifically aimed at enhancing EFs could be another important direction for future research. Currently, TMS protocols used in clinical practice and/or research are mainly derived from treatment protocols for related diseases, and the improvements or promotions in EFs may merely be secondary outcomes of the diagnostic and therapeutic processes. Future researchers could develop more targeted, personalized, safe, and tolerable TMS devices, protocols, or parameters based on the characteristics and needs of specific groups (such as children, the elderly, or those in professions like pilots and police), thereby facilitating the enhancement of EFs for the intended audiences (Antal et al. 2022). Furthermore, how to extend the intervention effects of TMS, that is, how to leverage limited intervention sessions to produce the longest possible beneficial impact on EFs of participants, might also be a matter that researchers must contemplate.

Further expanding the scope of clinical research is also one of the propositions that future researchers need to pay attention to, especially for mental disorders caused by neurological diseases. For example, vascular cognitive impairment (VCI), a clinical syndrome caused by cerebrovascular disease (Rundek et al. 2022), is often accompanied by executive dysfunctions (Levit, Hachinski, and Whitehead 2020). In this context, TMS has been widely applied both for diagnostic and therapeutic purposes (Lanza et al. 2017), as well as to monitor disease progression (Antczak, Rusin, and Slowik 2021). However, current research on the impacts of TMS on EFs in patients with VCI is still limited, and the reliability of such studies requires further validation (Wang and Dong 2024). For instance, stimulating the inferior frontal gyrus has been found to enhance EFs in patients with AD‐MCI (Li and Xiao 2024), whereas the study of Nicoletti et al. (2023) failed to observe any beneficial effects of HF‐TMS on EFs in patients with vascular depression. Considering the limited amount and small sample size of existing studies, it is therefore necessary to conduct more RCTs to verify the intervention effects of TMS on EFs in patients with VCI.

To enhance the effectiveness of promoting EFs, it may also be necessary to integrate TMS with other intervention methods and explore their mechanisms. For example, the LTP‐like effects induced by HF‐TMS primarily rely on the N‐methyl‐D‐aspartate receptors (NMDARs) on the postsynaptic membrane (Cirillo et al. 2017); therefore, combining HF‐TMS with oral NMDAR agonists (such as D‐cycloserine) could further enhance cortical excitability, thereby providing a possible means for promoting EFs (Kweon et al. 2023). In the future, exploring drugs or cognitive training that can synergistically enhance the effects of TMS may yield interesting outcomes for enhancing individual EFs.

## 6 Conclusions

In conclusion, research on TMS in the field of EFs has yielded certain charming results and holds a broad prospect of applications. Researchers should continue to explore the application potential of TMS for EFs and conduct more groundbreaking studies to investigate the mechanisms of EFs and to elevate the levels of EFs in both individuals and populations.

## Author Contributions

**Muyu Chen**: conceptualization, writing–review and editing, writing–original draft, software, investigation. **Guang Zhao**: writing–review and editing. **Li Peng**: conceptualization, writing–review and editing, investigation.

## Ethics Statement

The authors have nothing to report.

## Consent

The authors have nothing to report.

## Conflicts of Interest

The authors declare no conflicts of interest.

### Peer Review

The peer review history for this article is available at https://publons.com/publon/10.1002/brb3.70099.

## Data Availability

The authors have nothing to report.

## References

[brb370099-bib-0001] Ameis, S. H. , D. M. Blumberger , P. E. Croarkin , et al. 2020. “Treatment of Executive Function Deficits in Autism Spectrum Disorder With Repetitive Transcranial Magnetic Stimulation: A Double‐Blind, Sham‐Controlled, Pilot Trial.” Brain Stimulation 13: 539–547. 10.1016/j.brs.2020.01.007. 32289673 PMC8129776

[brb370099-bib-0002] Antal, A. , B. Luber , A. K. Brem , et al. 2022. “Non‐Invasive Brain Stimulation and Neuroenhancement.” Clinical Neurophysiology Practice 7: 146–165. 10.1016/j.cnp.2022.05.002. 35734582 PMC9207555

[brb370099-bib-0003] Antczak, J. , G. Rusin , and A. Slowik . 2021. “Transcranial Magnetic Stimulation as a Diagnostic and Therapeutic Tool in Various Types of Dementia.” Journal of Clinical Medicine 10: 2875. 10.3390/jcm10132875. 34203558 PMC8267667

[brb370099-bib-0004] Antunes, L. C. , J. L. Elkfury , C. S. Parizotti , et al. 2020. “Longer Cortical Silent Period Length Is Associated to Binge Eating Disorder: An Exploratory Study.” Frontiers in Psychiatry 11: 13. 10.3389/fpsyt.2020.559966. 33173510 PMC7591768

[brb370099-bib-0005] Barker, A. T. , R. Jalinous , and I. L. Freeston . 1985. “Non‐Invasive Magnetic Stimulation of Human Motor Cortex.” Lancet 1: 1106–1107. 10.1016/s0140-6736(85)92413-4. 2860322

[brb370099-bib-0006] Buhagiar, F. , M. Fitzgerald , J. Bell , F. Allanson , and C. Pestell . 2020. “Neuromodulation for Mild Traumatic Brain Injury Rehabilitation: A Systematic Review.” Frontiers in Human Neuroscience 14: 13. 10.3389/fnhum.2020.598208. 33362494 PMC7759622

[brb370099-bib-0007] Burke, M. J. , P. J. Fried , and A. Pascual‐Leone . 2019. “Transcranial Magnetic Stimulation: Neurophysiological and Clinical Applications.” Handbook of Clinical Neurology 163: 73–92. 10.1016/B978-0-12-804281-6.00005-7. 31590749

[brb370099-bib-0008] Cao, K. X. , M. L. Ma , C. Z. Wang , et al. 2021. “TMS‐EEG: An Emerging Tool to Study the Neurophysiologic Biomarkers of Psychiatric Disorders.” Neuropharmacology 197: 108574. 10.1016/j.neuropharm.2021.108574. 33894219

[brb370099-bib-0009] Cappon, D. , T. den Boer , C. Jordan , W. Yu , E. Metzger , and A. Pascual‐Leone . 2022. “Transcranial Magnetic Stimulation (TMS) for Geriatric Depression.” Ageing Research Reviews 74: 101531. 10.1016/j.arr.2021.101531. 34839043 PMC8996329

[brb370099-bib-0010] Cespon, J. , M. C. Pellicciari , E. P. Casula , and C. Miniussi . 2022. “Age‐Related Changes in Cortical Excitability Linked to Decreased Attentional and Inhibitory Control.” Neuroscience 495: 1–14. 10.1016/j.neuroscience.2022.05.021. 35605905

[brb370099-bib-0011] Chen, Y. , X. Li , L. Wang , et al. 2022. “Effects of Repetitive Transcranial Magnetic Stimulation on Cognitive Function in Patients with Stress‐Related Depression: A Randomized Double‐Blind fMRI and (1)H‐MRS Study.” Frontiers in Neurology 13: 844606. 10.3389/fneur.2022.844606. 35493813 PMC9051398

[brb370099-bib-0012] Chen, Y. H. , S. C. Liang , C. K. Sun , et al. 2023. “A Meta‐Analysis on the Therapeutic Efficacy of Repetitive Transcranial Magnetic Stimulation for Cognitive Functions in Attention‐Deficit/Hyperactivity Disorders.” BMC Psychiatry 23: 11. 10.1186/s12888-023-05261-2. 37845676 PMC10580630

[brb370099-bib-0013] Chou, Y. H. , M. Sundman , V. Ton That , J. Green , and C. Trapani . 2022. “Cortical Excitability and Plasticity in Alzheimer's Disease and Mild Cognitive Impairment: A Systematic Review and Meta‐Analysis of Transcranial Magnetic Stimulation Studies.” Ageing Research Reviews 79: 101660. 10.1016/j.arr.2022.101660. 35680080 PMC9707650

[brb370099-bib-0014] Chou, Y. H. , V. T. That , and M. Sundman . 2020. “A Systematic Review and Meta‐Analysis of rTMS Effects on Cognitive Enhancement in Mild Cognitive Impairment and Alzheimer's Disease.” Neurobiology of Aging 86: 1–10. 10.1016/j.neurobiolaging.2019.08.020. 31783330 PMC6995441

[brb370099-bib-0015] Chung, S. W. , C. J. Thomson , S. Lee , et al. 2019. “The Influence of Endogenous Estrogen on High‐Frequency Prefrontal Transcranial Magnetic Stimulation.” Brain Stimulation 12: 1271–1279. 10.1016/j.brs.2019.05.007. 31126870

[brb370099-bib-0016] Cirillo, G. , G. Di Pino , F. Capone , et al. 2017. “Neurobiological After‐Effects of Non‐Invasive Brain Stimulation.” Brain Stimulation 10: 1–18. 10.1016/j.brs.2016.11.009. 27931886

[brb370099-bib-0017] Corlier, J. , E. Burnette , A. C. Wilson , et al. 2020. “Effect of Repetitive Transcranial Magnetic Stimulation (rTMS) Treatment of Major Depressive Disorder (MDD) on Cognitive Control.” Journal of Affective Disorders 265: 272–277. 10.1016/j.jad.2020.01.068. 32090751

[brb370099-bib-0018] Cox, O. D. , A. Munjal , W. V. McCall , B. J. Miller , C. Baeken , and P. B. Rosenquist . 2023. “A Review of Clinical Studies of Electrodermal Activity and Transcranial Magnetic Stimulation.” Psychiatry Research 329: 115535. 10.1016/j.psychres.2023.115535. 37839318

[brb370099-bib-0019] Cristancho, P. , L. Kamel , M. Araque , et al. 2020. “iTBS to Relieve Depression and Executive Dysfunction in Older Adults: An Open Label Study.” American Journal of Geriatric Psychiatry 28: 1195–1199. 10.1016/j.jagp.2020.03.001. 32268978

[brb370099-bib-0020] Cristofori, I. , S. Cohen‐Zimerman , and J. Grafman . 2019. “Executive Functions.” Handbook of Clinical Neurology 163: 197–219. 10.1016/b978-0-12-804281-6.00011-2. 31590731

[brb370099-bib-0021] Dalton, B. , K. Foerde , S. Bartholdy , et al. 2020. “The Effect of Repetitive Transcranial Magnetic Stimulation on Food Choice‐Related Self‐Control in Patients With Severe, Enduring Anorexia Nervosa.” International Journal of Eating Disorders 53: 1326–1336. 10.1002/eat.23267. 32309882

[brb370099-bib-0022] Darmani, G. , T. O. Bergmann , K. Butts Pauly , et al. 2022. “Non‐Invasive Transcranial Ultrasound Stimulation for Neuromodulation.” Clinical Neurophysiology 135: 51–73. 10.1016/j.clinph.2021.12.010. 35033772

[brb370099-bib-0023] de Boer, N. S. , R. S. Schluter , J. G. Daams , Y. D. van der Werf , A. E. Goudriaan , and R. J. van Holst . 2021. “The Effect of Non‐Invasive Brain Stimulation on Executive Functioning in Healthy Controls: A Systematic Review and Meta‐Analysis.” Neuroscience & Biobehavioral Reviews 125: 122–147. 10.1016/j.neubiorev.2021.01.013. 33503477

[brb370099-bib-0024] Deng, X. P. , J. Wang , Y. F. Zang , et al. 2022. “Intermittent Theta Burst Stimulation Over the Parietal Cortex Has a Significant Neural Effect on Working Memory.” Human Brain Mapping 43: 1076–1086. 10.1002/hbm.25708. 34730863 PMC8764471

[brb370099-bib-0025] Diamond, A. 2013. “Executive Functions.” Annual Review of Psychology 64: 135–168. 10.1146/annurev-psych-113011-143750. PMC4084861 23020641

[brb370099-bib-0026] Eysel, U. T. , and D. Jancke . 2024. “Induction of Excitatory Brain State Governs Plastic Functional Changes in Visual Cortical Topology.” Brain Structure and Function 229: 531–547. 10.1007/s00429-023-02730-y. 38041743 PMC10978694

[brb370099-bib-0027] Fisicaro, F. , G. Lanza , R. Bella , and M. Pennisi . 2020. “Self‐Neuroenhancement: The Last Frontier of Noninvasive Brain Stimulation?” Journal of Clinical Neurology 16: 158–159. 10.3988/jcn.2020.16.1.158. 31942774 PMC6974812

[brb370099-bib-0028] Fitzsimmons, S. M. D. D. , Y. D. van der Werf , A. D. van Campen , et al. 2022. “Repetitive Transcranial Magnetic Stimulation for Obsessive‐Compulsive Disorder: A Systematic Review and Pairwise/Network Meta‐Analysis.” Journal of Affective Disorders 302: 302–312. 10.1016/j.jad.2022.01.048. 35041869

[brb370099-bib-0029] Friedman, N. P. , and T. W. Robbins . 2022. “The Role of Prefrontal Cortex in Cognitive Control and Executive Function.” Neuropsychopharmacology 47: 72–89. 10.1038/s41386-021-01132-0. 34408280 PMC8617292

[brb370099-bib-0030] Friehs, M. A. , J. Siodmiak , M. C. Donzallaz , et al. 2023. “No Effects of 1 Hz Offline TMS on Performance in the Stop‐Signal Game.” Scientific Reports 13: 12. 10.1038/s41598-023-38841-z. 37463991 PMC10354051

[brb370099-bib-0031] Ganesan, K. , and N. Steinbeis . 2022. “Development and Plasticity of Executive Functions: A Value‐Based Account.” Current Opinion in Psychology 44: 215–219. 10.1016/j.copsyc.2021.09.012. 34717277

[brb370099-bib-0032] Gao, Y. , Y. Qiu , Q. Y. Yang , et al. 2023. “Repetitive Transcranial Magnetic Stimulation Combined With Cognitive Training for Cognitive Function and Activities of Daily Living in Patients With Post‐Stroke Cognitive Impairment: A Systematic Review and Meta‐Analysis.” Ageing Research Reviews 87: 12. 10.1016/j.arr.2023.101919. 37004840

[brb370099-bib-0033] Han, X. X. , S. J. Cai , H. Gui , and R. Chen . 2023. “Transcranial Magnetic Stimulation Cortical Oscillations and Improve Cognition in Obstructive Sleep Apnea Patients.” Brain and Behavior 13: 10. 10.1002/brb3.2958. PMC10097149 36922909

[brb370099-bib-0034] Harmelech, T. , C. A. Hanlon , and A. Tendler . 2023. “Transcranial Magnetic Stimulation as a Tool to Promote Smoking Cessation and Decrease Drug and Alcohol Use.” Brain Sciences 13: 15. 10.3390/brainsci13071072. PMC10377606 37509004

[brb370099-bib-0035] Hayashi, C. Y. , I. S. Neville , P. A. Rodrigues , et al. 2018. “Altered Intracortical Inhibition in Chronic Traumatic Diffuse Axonal Injury.” Frontiers in Neurology 9: 8. 10.3389/fneur.2018.00189. 29643831 PMC5882787

[brb370099-bib-0036] He, P. K. , L. M. Wang , J. N. Chen , et al. 2022. “Repetitive Transcranial Magnetic Stimulation (rTMS) Fails to Improve Cognition in Patients With Parkinson's Disease: A Meta‐Analysis of Randomized Controlled Trials.” International Journal of Neuroscience 132: 269–282. 10.1080/00207454.2020.1809394. 33208009

[brb370099-bib-0037] Hernandez‐Pavon, J. C. , D. Veniero , T. O. Bergmann , et al. 2023. “TMS Combined With EEG: Recommendations and Open Issues for Data Collection and Analysis.” Brain Stimulation 16: 567–593. 10.1016/j.brs.2023.02.009. 36828303

[brb370099-bib-0038] Hong, H. , R. Y. Kim , Y. Song , et al. 2022. “Genetic Profile for Dopamine Signaling Predicts Brain Functional Reactivity to Repetitive Transcranial Magnetic Stimulation.” European Archives of Psychiatry and Clinical Neuroscience 273: 13. 10.1007/s00406-022-01436-2. 35951113

[brb370099-bib-0039] Horowitz‐Kraus, T. , K. Randell , and I. Morag . 2023. “Neurobiological Perspective on the Development of Executive Functions.” Acta Paediatrica 112: 1860–1864. 10.1111/apa.16883. 37338188

[brb370099-bib-0040] Hsu, C. W. , P. H. Chou , A. R. Brunoni , et al. 2024. “Comparing Different Non‐Invasive Brain Stimulation Interventions for Bipolar Depression Treatment: A Network Meta‐Analysis of Randomized Controlled Trials.” Neuroscience and Biobehavioral Reviews 156: 105483. 10.1016/j.neubiorev.2023.105483. 38056187

[brb370099-bib-0041] Jones, D. T. , and J. Graff‐Radford . 2021. “Executive Dysfunction and the Prefrontal Cortex.” Continuum 27: 1586–1601. 10.1212/CON.0000000000001009. 34881727

[brb370099-bib-0042] Kaller, C. P. , K. Heinze , A. Frenkel , et al. 2013. “Differential Impact of Continuous Theta‐Burst Stimulation Over Left and Right DLPFC on Planning.” Human Brain Mapping 34: 36–51. 10.1002/hbm.21423. 22002416 PMC6870309

[brb370099-bib-0043] Kamble, N. , M. Netravathi , B. C. Nagaraju , et al. 2018. “Evaluation of Cognition and Cortical Excitability in Huntington's Disease.” Canadian Journal of Neurological Sciences 45: 176–181. 10.1017/cjn.2017.277. 29307324

[brb370099-bib-0044] Kan, R. L. D. , F. Padberg , C. G. Giron , et al. 2023. “Effects of Repetitive Transcranial Magnetic Stimulation of the Left Dorsolateral Prefrontal Cortex on Symptom Domains in Neuropsychiatric Disorders: A Systematic Review and Cross‐Diagnostic Meta‐Analysis.” Lancet Psychiatry 10: 252–259. 10.1016/s2215-0366(23)00026-3. 36898403

[brb370099-bib-0045] Kirkovski, M. , P. H. Donaldson , M. Do , et al. 2023. “A Systematic Review of the Neurobiological Effects of Theta‐Burst Stimulation (TBS) as Measured Using Functional Magnetic Resonance Imaging (fMRI).” Brain Structure and Function 228: 717–749. 10.1007/s00429-023-02634-x. 37072625 PMC10113132

[brb370099-bib-0046] Kiyonaga, A. , J. P. Powers , Y. C. Chiu , and T. Egner . 2021. “Hemisphere‐Specific Parietal Contributions to the Interplay Between Working Memory and Attention.” Journal of Cognitive Neuroscience 33: 1428–1441. 10.1162/jocn_a_01740. 34496381 PMC9422895

[brb370099-bib-0047] Kumar, S. , R. Zomorrodi , Z. Ghazala , et al. 2023. “Effects of Repetitive Paired Associative Stimulation on Brain Plasticity and Working Memory in Alzheimer's Disease: A Pilot Randomized Double‐Blind‐Controlled Trial.” International Psychogeriatrics 35: 143–155. 10.1017/s1041610220003518. 33190659

[brb370099-bib-0048] Kweon, J. , M. M. Vigne , R. N. Jones , L. L. Carpenter , and J. C. Brown . 2023. “Practice Makes Plasticity: 10‐Hz rTMS Enhances LTP‐Like Plasticity in Musicians and Athletes.” Frontiers in Neural Circuits 17: 1124221. 10.3389/fncir.2023.1124221. 37025991 PMC10070804

[brb370099-bib-0049] Lanza, G. , P. Bramanti , M. Cantone , M. Pennisi , G. Pennisi , and R. Bella . 2017. “Vascular Cognitive Impairment Through the Looking Glass of Transcranial Magnetic Stimulation.” Behavioural Neurology 2017: 1421326. 10.1155/2017/1421326. 28348458 PMC5350538

[brb370099-bib-0050] Lanza, G. , F. Fisicaro , M. Cantone , et al. 2023. “Repetitive Transcranial Magnetic Stimulation in Primary Sleep Disorders.” Sleep Medicine Reviews 67: 101735. 10.1016/j.smrv.2022.101735. 36563570

[brb370099-bib-0051] Lefaucheur, J. P. 2019. “Transcranial Magnetic Stimulation.” Handbook of Clinical Neurology 160: 559–580. 10.1016/B978-0-444-64032-1.00037-0. 31277876

[brb370099-bib-0052] Lefaucheur, J. P. , A. Aleman , C. Baeken , et al. 2020. “Evidence‐Based Guidelines on the Therapeutic Use of Repetitive Transcranial Magnetic Stimulation (rTMS): An Update (2014–2018).” Clinical Neurophysiology 131: 474–528. 10.1016/j.clinph.2019.11.002. 31901449

[brb370099-bib-0053] Levit, A. , V. Hachinski , and S. N. Whitehead . 2020. “Neurovascular Unit Dysregulation, White Matter Disease, and Executive Dysfunction: The Shared Triad of Vascular Cognitive Impairment and Alzheimer Disease.” GeroScience 42: 445–465. 10.1007/s11357-020-00164-6. 32002785 PMC7205943

[brb370099-bib-0054] Li, C.‐T. , C.‐H. Juan , H.‐C. Lin , et al. 2022. “Cortical Excitatory and Inhibitory Correlates of the Fronto‐Limbic Circuit in Major Depression and Differential Effects of Left Frontal Brain Stimulation in a Randomized Sham‐Controlled Trial.” Journal of Affective Disorders 311: 364–370. 10.1016/j.jad.2022.05.107. 35618168

[brb370099-bib-0055] Li, S. , J. N. Jin , X. Wang , H. Z. Qi , Z. P. Liu , and T. Yin . 2017. “Theta and Alpha Oscillations During the Retention Period of Working Memory by rTMS Stimulating the Parietal Lobe.” Frontiers in Behavioral Neuroscience 11: 12. 10.3389/fnbeh.2017.00170. 28959194 PMC5603655

[brb370099-bib-0056] Li, S. , and Z. Xiao . 2024. “Recent Research Progress on the Use of Transcranial Magnetic Stimulation in the Treatment of Vascular Cognitive Impairment.” Neuropsychiatric Disease and Treatment 20: 1235–1246. 10.2147/ndt.S467357. 38883416 PMC11179638

[brb370099-bib-0057] Liu, S. S. , X. L. Wang , J. Q. Ma , et al. 2021. “Effect of Low‐Frequency Repetitive Transcranial Magnetic Stimulation on Executive Function and Its Neural Mechanism: An Event‐Related Potential Study.” Frontiers in neuroscience 15: 10. 10.3389/fnins.2021.701560. PMC8580383 34776839

[brb370099-bib-0058] Liu, Y. , J. Luo , J. Fang , et al. 2023. “Screening Diagnosis of Executive Dysfunction After Ischemic Stroke and the Effects of Transcranial Magnetic Stimulation: A Prospective Functional Near‐Infrared Spectroscopy Study.” CNS neuroscience & therapeutics 29: 1561–1570. 10.1111/cns.14118. 36786133 PMC10173709

[brb370099-bib-0059] Luciana, M. , and P. F. Collins . 2022. “Neuroplasticity, the Prefrontal Cortex, and Psychopathology‐Related Deviations in Cognitive Control.” Annual Review of Clinical Psychology 18: 443–469. 10.1146/annurev-clinpsy-081219-111203. 35534121

[brb370099-bib-0060] Martin, D. M. , S. M. McClintock , J. Forster , and C. K. Loo . 2016. “Does Therapeutic Repetitive Transcranial Magnetic Stimulation Cause Cognitive Enhancing Effects in Patients With Neuropsychiatric Conditions? A Systematic Review and Meta‐Analysis of Randomised Controlled Trials.” Neuropsychology Review 26: 295–309. 10.1007/s11065-016-9325-1. 27632386 PMC5949868

[brb370099-bib-0061] Mizutani‐Tiebel, Y. , M. Tik , K. Y. Chang , et al. 2022. “Concurrent TMS‐fMRI: Technical Challenges, Developments, and Overview of Previous Studies.” Front Psychiatry 13: 825205. 10.3389/fpsyt.2022.825205. 35530029 PMC9069063

[brb370099-bib-0062] Morgan, H. M. , M. C. Jackson , M. G. van Koningsbruggen , K. L. Shapiro , and D. E. J. Linden . 2013. “Frontal and Parietal Theta Burst TMS Impairs Working Memory for Visual‐Spatial Conjunctions.” Brain Stimulation 6: 122–129. 10.1016/j.brs.2012.03.001. 22483548 PMC3605569

[brb370099-bib-0063] Muhle‐Karbe, P. S. , J. F. Jiang , and T. Egner . 2018. “Causal Evidence for Learning‐Dependent Frontal Lobe Contributions to Cognitive Control.” Journal of Neuroscience 38: 962–973. 10.1523/jneurosci.1467-17.2017. 29229706 PMC5783969

[brb370099-bib-0064] Nardone, R. , L. Sebastianelli , V. Versace , et al. 2020. “Effects of Repetitive Transcranial Magnetic Stimulation in Subjects With Sleep Disorders.” Sleep Medicine 71: 113–121. 10.1016/j.sleep.2020.01.028. 32173186

[brb370099-bib-0065] Newman‐Norlund, R. D. , M. Gibson , P. A. McConnell , and B. Froeliger . 2020. “Dissociable Effects of Theta‐Burst Repeated Transcranial Magnetic Stimulation to the Inferior Frontal Gyrus on Inhibitory Control in Nicotine Addiction.” Frontiers in Psychiatry 11: 8. 10.3389/fpsyt.2020.00260. 32351412 PMC7174714

[brb370099-bib-0066] Nicoletti, V. G. , F. Fisicaro , E. Aguglia , et al. 2023. “Challenging the Pleiotropic Effects of Repetitive Transcranial Magnetic Stimulation in Geriatric Depression: A Multimodal Case Series Study.” Biomedicines 11: 958. 10.3390/biomedicines11030958. 36979937 PMC10046045

[brb370099-bib-0067] Noda, Y. , M. S. Barr , R. Zomorrodi , et al. 2018. “Reduced Short‐Latency Afferent Inhibition in Prefrontal but Not Motor Cortex and Its Association with Executive Function in Schizophrenia: A Combined TMS‐EEG Study.” Schizophrenia Bulletin 44: 193–202. 10.1093/schbul/sbx041. 28379529 PMC5768054

[brb370099-bib-0068] Nursey, J. , A. Sbisa , H. Knight , et al. 2020. “Exploring Theta Burst Stimulation for Post‐Traumatic Stress Disorder in Australian Veterans—A Pilot Study.” Military Medicine 185: E1770–E1778. 10.1093/milmed/usaa149. 32601710

[brb370099-bib-0069] Osada, T. , S. Ohta , A. Ogawa , et al. 2019. “An Essential Role of the Intraparietal Sulcus in Response Inhibition Predicted by Parcellation‐Based Network.” Journal of Neuroscience 39: 2509–2521. 10.1523/jneurosci.2244-18.2019. 30692225 PMC6435821

[brb370099-bib-0070] Parris, B. A. , M. G. Wadsley , G. Arabaci , N. Hasshim , M. Augustinova , and L. Ferrand . 2021. “The Effect of High‐Frequency rTMS of the Left Dorsolateral Prefrontal Cortex on the Resolution of Response, Semantic and Task Conflict in the Colour‐Word Stroop Task.” Brain Structure and Function 226: 1241–1252. 10.1007/s00429-021-02237-4. 33608822 PMC8036200

[brb370099-bib-0071] Polania, R. , M. A. Nítsche , and C. C. Ruff . 2018. “Studying and Modifying Brain Function With Non‐Invasive Brain Stimulation.” Nature Neuroscience 21: 174–187. 10.1038/s41593-017-0054-4. 29311747

[brb370099-bib-0072] Pulopulos, M. M. , J. Allaert , M. A. Vanderhasselt , et al. 2022. “Effects of HF‐rTMS Over the Left and Right DLPFC on Proactive and Reactive Cognitive Control.” Social Cognitive and Affective Neuroscience 17: 109–119. 10.1093/scan/nsaa082. 32613224 PMC8824550

[brb370099-bib-0073] Quoilin, C. , E. Wilhelm , P. Maurage , P. de Timary , and J. Duque . 2018. “Deficient Inhibition in Alcohol‐Dependence: Let's Consider the Role of the Motor System!.” Neuropsychopharmacology 43: 1851–1858. 10.1038/s41386-018-0074-0. 29728650 PMC6046042

[brb370099-bib-0074] Rostami, R. , R. Kazemi , Z. Nasiri , S. Ataei , A. L. Hadipour , and N. Jaafari . 2022. “Cold Cognition as Predictor of Treatment Response to rTMS; A Retrospective Study on Patients With Unipolar and Bipolar Depression.” Frontiers in Human Neuroscience 16: 13. 10.3389/fnhum.2022.888472. PMC9358278 35959241

[brb370099-bib-0075] Rozov, A. V. , F. F. Valiullina , and A. P. Bolshakov . 2017. “Mechanisms of Long‐Term Plasticity of Hippocampal GABAergic Synapses.” Biochemistry 82: 257–263. 10.1134/s0006297917030038. 28320266

[brb370099-bib-0076] Rundek, T. , M. Tolea , T. Ariko , E. A. Fagerli , and C. J. Camargo . 2022. “Vascular Cognitive Impairment (VCI).” Neurotherapeutics 19: 68–88. 10.1007/s13311-021-01170-y. 34939171 PMC9130444

[brb370099-bib-0077] Salehinejad, M. A. , E. Ghanavati , M. H. A. Rashid , and M. A. Nitsche . 2021. “Hot and Cold Executive Functions in the Brain: A Prefrontal‐Cingular Network.” Brain and Neuroscience Advances 5: 23982128211007769. 10.1177/23982128211007769. 33997292 PMC8076773

[brb370099-bib-0078] Schulze, L. , S. Wheeler , M. P. McAndrews , C. J. E. Solomon , P. Giacobbe , and J. Downar . 2016. “Cognitive Safety of Dorsomedial Prefrontal Repetitive Transcranial Magnetic Stimulation in Major Depression.” European Neuropsychopharmacology 26: 1213–1226. 10.1016/j.euroneuro.2016.04.004. 27157074

[brb370099-bib-0079] Sciortino, D. , A. Pigoni , G. Delvecchio , E. Maggioni , G. Schiena , and P. Brambilla . 2021. “Role of rTMS in the Treatment of Cognitive Impairments in Bipolar Disorder and Schizophrenia: A Review of Randomized Controlled Trials.” Journal of Affective Disorders 280: 148–155. 10.1016/j.jad.2020.11.001. 33212406

[brb370099-bib-0080] Siebner, H. R. , K. Funke , A. S. Aberra , et al. 2022. “Transcranial Magnetic Stimulation of the Brain: What Is Stimulated?—A Consensus and Critical Position Paper.” Clinical Neurophysiology 140: 59–97. 10.1016/j.clinph.2022.04.022. 35738037 PMC9753778

[brb370099-bib-0081] Sun, W. , Q. Wu , L. Gao , et al. 2023. “Advancements in Transcranial Magnetic Stimulation Research and the Path to Precision.” Neuropsychiatric Disease and Treatment 19: 1841–1851. 10.2147/NDT.S414782. 37641588 PMC10460597

[brb370099-bib-0082] Suppa, A. , F. Asci , and A. Guerra . 2022. “Transcranial Magnetic Stimulation as a Tool to Induce and Explore Plasticity in Humans.” Handbook of Clinical Neurology 184: 73–89. 10.1016/B978-0-12-819410-2.00005-9. 35034759

[brb370099-bib-0083] Tulviste, J. , E. Goldberg , K. Podell , M. Vaht , J. Harro , and T. Bachmann . 2019. “BDNF Polymorphism in Non‐Veridical Decision Making and Differential Effects of rTMS.” Behavioural Brain Research 364: 177–182. 10.1016/j.bbr.2019.02.027. 30776391

[brb370099-bib-0084] Viñas‐Guasch, N. , T. H. B. Ng , J. G. Heng , et al. 2022. “Cerebellar Transcranial Magnetic Stimulation (TMS) Impairs Visual Working Memory.” Cerebellum 22: 16. 10.1007/s12311-022-01396-2. PMC9522915 35355219

[brb370099-bib-0085] Wang, K. , and Q. Dong . 2024. “Chinese Guidelines for the Diagnosis and Treatment of Vascular Cognitive Impairment (2024 edition).” Chinese Medical Journal 104: 1–14. 10.3760/cma.j.cn112137-20240501-01024 38866700

[brb370099-bib-0086] Wang, Y. 2024. “Chinese Guideline for Diagnosis and Treatment of Insomnia (2023).” Chinese Journal of Neurology 57: 560–584. 10.3760/cma.j.cn113694-20240406-00209.

[brb370099-bib-0087] Webler, R. D. , J. Fox , L. M. McTeague , et al. 2022. “DLPFC Stimulation Alters Working Memory Related Activations and Performance: An Interleaved TMS‐fMRI Study.” Brain Stimulation 15: 823–832. 10.1016/j.brs.2022.05.014. 35644517

[brb370099-bib-0088] Wexler, A. 2022. “Mapping the Landscape of Do‐It‐Yourself Medicine.” Citizen Science: Theory and Practice 7: 38. 10.5334/cstp.553. 36632334 PMC9830450

[brb370099-bib-0089] Wu, Q. L. , Y. Gao , J. T. Li , W. Y. Ma , and N. H. Chen . 2022. “The Role of AMPARs Composition and Trafficking in Synaptic Plasticity and Diseases.” Cellular and Molecular Neurobiology 42: 2489–2504. 10.1007/s10571-021-01141-z. 34436728 PMC11421597

[brb370099-bib-0090] Xu, M. , S. Nikolin , N. Samaratunga , E. J. H. Chow , C. K. Loo , and D. M. Martin . 2023. “Cognitive Effects Following Offline High‐Frequency Repetitive Transcranial Magnetic Stimulation (HF‐rTMS) in Healthy Populations: A Systematic Review and Meta‐Analysis.” Neuropsychology Review 34: 27. 10.1007/s11065-023-09580-9. PMC10920443 36857011

[brb370099-bib-0091] Yue, Q. H. , and R. C. Martin . 2022. “Phonological Working Memory Representations in the Left Inferior Parietal Lobe in the Face of Distraction and Neural Stimulation.” Frontiers in Human Neuroscience 16: 18. 10.3389/fnhum.2022.890483. PMC9259857 35814962

[brb370099-bib-0092] Zhang, B. B. B. , R. L. D. Kan , C. G. Giron , T. T. Z. Lin , S. Y. Yau , and G. S. Kranz . 2022. “Dose‐Response Relationship Between iTBS and Prefrontal Activation During Executive Functioning: A fNIRS Study.” Frontiers in Psychiatry 13: 12. 10.3389/fpsyt.2022.1049130. PMC9807664 36606127

[brb370099-bib-0093] Zhang, R. , and N. D. Volkow . 2019. “Brain Default‐Mode Network Dysfunction in Addiction.” Neuroimage 200: 313–331. 10.1016/j.neuroimage.2019.06.036. 31229660

[brb370099-bib-0094] Zhang, T. H. , M. M. Guo , G. Z. Xu , L. H. Ji , and Z. H. Wang . 2022. “Effect of Intermittent Theta Burst Stimulation on Cross‐Brain Neural Network Between the Hippocampus and Prefrontal Cortex of Working Memory in Rats.” Progress in Biochemistry and Biophysics 49: 1573–1585. 10.16476/j.pibb.2021.0243.

[brb370099-bib-0095] Zuk, P. , L. Torgerson , D. Sierra‐Mercado , and G. Lázaro‐Muñoz . 2018. “Neuroethics of Neuromodulation: An Update.” Current Opinion in Biomedical Engineering 8: 45–50. 10.1016/j.cobme.2018.10.003. 30687802 PMC6345549

